# Consumption: Its Cause and Nature

**Published:** 1890-05

**Authors:** 


CONSUMPTION: ITS CAUSE AND NATURE, by Rollin R. Gregg, M. D., to which is added THE THERAPEUTICS OF TUBERCULOUS AFFECTIONS, by H. C. Allen, M. D. Ann Arbor, Michigan, 1889.

When it was announced that this work was to be after the plan of Bell on *Diarrhœa and Dysentery*, we looked forward to possessing a book that would do as much for us in that scourge, consumption, as Bell's work does for us in treating morbid discharges from the bowels. We regret to say we are disappointed.

In the first part of the work is given Dr. Gregg's pet theory: that this and other affections are due to a loss of albumen through diseased mucous membranes.

Although this is elaborately set forth, and many authors quoted to bolster up this idea, we are obliged to say that it is but theory, and the arguments advanced are not convincing. While it is well known that there is great loss of albumen in this malady, as well as in many others, if we were to give any theorizing to the subject, we should ask: Why this loss? Dr. Gregg says because of the "diseased mucous membranes." But the why of the diseased mucous membranes is the pertinent and important question. When we reach this point we are approaching a question that is not answerable. Certainly it is not answered on p. 58, where the author says: "Whatever else the consumptive may have inherited of actual disease, or a tendency to it, he inherits a characteristic weakness of the mucous membranes, or a great liability to catarrhal irritations arising therein upon slight provocations, from moderate as well as from severe colds, and the like. . . ."

"Secondly. When chronic skin diseases are treated locally, and thereby removed from the surface, no other result follows, except that they are driven inwardly to locate upon the mucous membranes, where they go into immediate chronic catarrhal disease. . . ." All this is true of some cases, but a very large number of consumptives never present any skin diseases, and in many catarrhal affections do not appear until the deposition of tubercles. We can see the theory may hold good as to some, but it has no relation whatever to others. Indeed the author himself casts doubt upon the whole question, at p. 169, in these words: "If the cause of consumption is the loss of albumen from the blood through irritated and abraded mucous membranes, as must be conceded on the evidence presented to be at least highly probable, then the curing of the cause—that is, properly healing all the mucous surfaces that are diseased—must of a necessity put a stop to the further production of tubercles and the whole category of attending conditions and sufferings. Of this there can be no reasonable question, provided always, of course, that the real cause has been fathomed and is the loss of albumen as described." If we had been impressed with the theory before reading this paragraph we certainly should begin to doubt its correctness after reading it.

Fortunately—thanks to the genius of Hahnemann—we need pay little attention to any theory regarding the nature of disease. Simply, we can *know* nothing about it. While we may be familiar with the structural changes caused by disease, when we approach the problem of the cause and nature of the various maladies we advance to an insolvable problem.

When we come to the part of the work which gives us the therapeutics we can have no doubt. No one who has put Hahnemannian Homœopathy to the test need have any uncertainty here.

As we have said above, we are disappointed in this work. Not for what is given, however, but for the omissions. We feel that the editor of the work has not furnished all that could be had ready to hand for the taking. We are thankful for what he has done, and we trust that at no distant day he will go farther and give us more. G. H. C.

